# Prostate cancer stem cells and their targeted therapies

**DOI:** 10.3389/fcell.2024.1410102

**Published:** 2024-08-08

**Authors:** Huilan Su, Liqun Huang, Jianjun Zhou, Guosheng Yang

**Affiliations:** ^1^ Research Center for Translational Medicine, Cancer Stem Cell Institute, Shanghai East Hospital, Tongji University School of Medicine, Shanghai, China; ^2^ Department of Urology, Shanghai East Hospital, Tongji University School of Medicine, Shanghai, China

**Keywords:** prostate cancer, prostate cancer stem cells, basal progenitor cells, luminal progenitor cells, targeted therapy

## Abstract

Prostate cancer (PCa) is the most common malignancy among men worldwide. Through androgen receptor signaling inhibitor (ARSI) treatment, patients eventually succumb to castration-resistant prostate cancer (CRPC). For this, the prostate cancer stem cells (PCSCs), as a minor population of tumor cells that can promote tumor relapse, ARSI resistance, and disease progression, are gaining attention. Therefore, specific therapy targeting PCSCs has momentum. This study reviewed the identification and characterization of PCSCs and PCSC-based putative biomarkers and summarized their mechanisms of action. We further discussed clinical trials of novel therapeutic interventions focused on PCSC-related pathways, the PCSC microenvironment, cutting-edge miRNA therapy, and immunotherapy approaches from a mechanistic standpoint. This review provides updated insights into PCSC plasticity, identifying new PCSC biomarkers and optimized treatments for patients with advanced PCa.

## Graphical Abstract

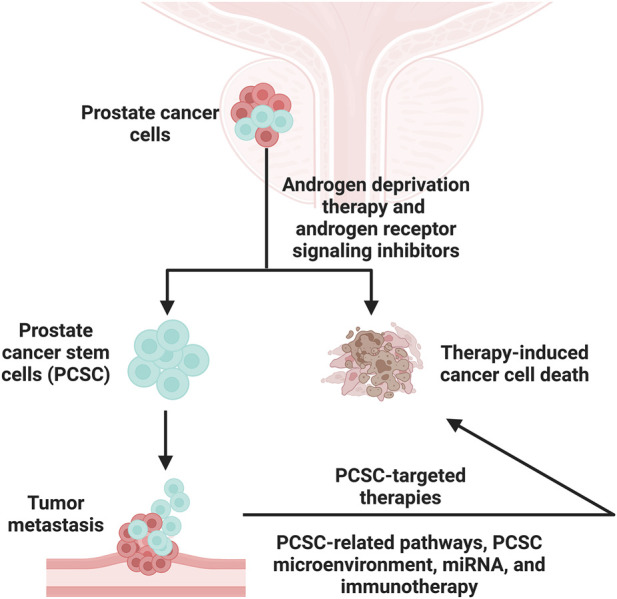

## 1 Introduction

Prostate cancer (PCa) is the most common malignancy among men, accounting for 29% of estimated new cancer cases (2023) and a substantial burden to the public health system (Porcacchia et al., 2022; Siegel et al., 2023). Worldwide, PCa ranks second in terms of mortality (400,000 deaths annually), and this rate is expected to be 2-fold higher by 2040 (Sandhu et al., 2021). Even in an East Asian country like China, where the incidence rate of prostate cancer is generally low, the number of PCa patients is continually on the rise, making it a front-runner of urinary tumor-related disease (Qiu et al., 2021). As the disease progresses, PCa might undergo a transition phase from hormone-sensitive prostate cancer (HSPC) to castration-resistant prostate cancer (CRPC) and from localized disease to metastatic castration-resistant prostate cancer (mCRPC) (Terrisse et al., 2022; Yehya et al., 2022). When diagnosed with an advanced stage, men would have a considerably diminished 5-year overall survival (OS) (30%), making advanced PCa a threat to patients (Gao et al., 2020).

The onset and progression of PCa are driven by androgen receptor (AR) signaling (Zheng et al., 2022). However, despite being initially effective and durable for localized and advanced prostate tumors, androgen deprivation therapy (ADT) and AR-directed strategies (e.g., enzalutamide) will move to a stage characterized by the inevitable emergence of resistance (Storck et al., 2022; Zheng et al., 2022; Zhu et al., 2022). At this point, the heterogeneous progenies containing enriched PCSCs from advanced PCa become the predominant population and are almost all negative in prostate-specific antigen (PSA) and AR levels (Zhang et al., 2015).

How does the unique mechanism contribute to treatment resistance? The inherent properties of prostate cancer stem cells (PCSC) may provide new insights into this puzzle. We identified that AR^−/lo^ cells were linked to increased PCSC populations, which are proven to promote tumor relapse and disease progression (Tang, 2022). Accordingly, understanding the molecular features of the PCSCs that drive the phenotypic transition from ADT-sensitivity to CRPC could help provide more meaningful results for ongoing research and designing more appropriate treatment strategies in the clinic.

## 2 Presence of PCSC

A prerequisite to tracking PCSCs is enumerating normal human prostate (NHP) cell lineages in full detail. Given their histological appearance and specific antigen expression, the epithelial cells of NHP are composed of basal and luminal layers and scarce neuroendocrine (NE) cells (Tang, 2022). The basal layer contains a small population of multipotent stem cells (SCs) (<5%), whereas the number in the luminal layer is less than 1% (Abate-Shen and Shen, 2000). Investigating the unique properties of layers would be meaningful for understanding and laying a foundation for novel therapeutics targeting PCSCs.

### 2.1 Basal cells

Owing to the relative undifferentiation and survival priority of AR ablation, the basal population of epithelial cells tends to possess characterizations of PCSCs (Goldstein et al., 2008). In addition, basal cells preferentially express cell adhesion/cytoskeleton and extracellular matrix remodeling-related genes (Zhang D. et al., 2016). Lineage-tracing studies revealed that PTEN deletion and deacetylated Klf5 contributed to rapid differentiation of luminal progeny by controlling basal progenitor cell fate (Zhang J. et al., 2018; Zhang et al., 2020). Interestingly, acute prostatitis mediated the differentiation of basal cells into luminal cells via a specific program in the microenvironment (Toivanen et al., 2016). Meanwhile, Kwon et al. (2014) described a mouse model where tissue repair in the prostate epithelium was regulated partly by basal-to-luminal differentiation. In addition, basal cells functionally revealing neurogenic properties brought out the underlying hypothesis of cells-or-origin for neuroendocrine prostate cancer (NEPC) (Verma et al., 2023). Of clinical relevance, Zhang et al. revealed the contribution of basal cells to promote castration-resistant and metastatic PCa (Zhang D. et al., 2016). Multiple crucial molecules such as CK14, B-cell lymphoma-2 (Bcl-2), and human telomerase reverse transcriptase (hTERT) have been well documented to preferentially localize in the basal layer (Wang et al., 2009; Banerjee et al., 2023). Thus, it is thought that basal cells share SC characteristics. On the other hand, a growing body of evidence has increasingly linked SC-like cells to the basal cells due to their co-expressed markers. *In vitro* and *in vivo* prostate assays have exhibited SC-enhanced global transcription and rRNA transcription activity in the basal layer (Zhang D. et al., 2016). Many other protein markers associated with CSC phenotype, such as spinocerebellar ataxia 1 (Sca-1), CD133, CD44, CD117, CD49f, α2 integrin, C-X-C motif chemokine receptor type 4 (CXCR4), epithelial cell adhesion molecule (EpCaM), CD54, and sex-determining region Y-box 2 (SOX2) et al., have also been detected in basal cells (details are shown in Table1) (Goldstein et al., 2008; Wang et al., 2009; Hoogland et al., 2014; Galoczova et al., 2021; Verma et al., 2023). Tumor samples derived from Sca-1, CD133, CD44, and CD117-positive cells basal cells possessed the self-renewal ability and reconstituted the prostatic ducts (Wang et al., 2009). However, Hoogland et al. (2014) raised doubt about the reliability of CD117, CD133, and OCT3/4 to label PCSC characteristics because these markers were not detected in clinical tissue. For specific treatment, ADT-treated PCa tended to develop into NEPC, accompanied by high levels of stem- (SOX2) and basal cell markers (KRT5; TP63) (Verma et al., 2023). This could provide a novel platform for screening drug candidates in a clinical situation via monitoring the ADT-resistant stem cell-like population.

**TABLE 1:** List of putative biomarkers for CSCs.

Heterogeneity markers	Localization	Details of stem-like characteristics
CD49f	Basal cells (Tang, 2022)	The basal stem cells express high levels of CD49f (integrin α6), CD133, and Bcl-2 (Tang, 2022)
CD133		
Bcl-2		
KRT16/17/6	Basal cells (Hu et al., 2021)	Single-cell RNA-seq analysis reveals prostate active stem cells and bipotent progenitor cells, keratin16/17/6 (KRT16/17/6), are enriched (Hu et al., 2021)
CK14	Basal cells (Tang, 2022)	The basal cell layer consists of differentiated CK5^+^/CK14^+^/p63^+^ basal stem cells (Tang, 2022)
p63		
hTERT	Basal cells (Banerjee et al., 2023)	The high hTERT prostate cancer cells exhibit CSC properties (Zhang et al., 2017)
EpCaM	Basal cells (Mohtar et al., 2020)	EpCaM-specific chimeric antigen receptors enable them to target the CSC marker EpCaM (CD326) (Deng et al., 2015)
CXCR4	Basal cells (Darash-Yahana et al., 2004)	Activated platelets secrete stromal-derived growth factor-1α (SDF-1α) and can mobilize CSCs via the CXCR4 receptor (Rudzinski et al., 2021)
CD54	Basal cells (Li et al., 2017a)	CD54 (ICAM1) could be a novel, reliable prostate CSC marker (Li et al., 2017a)
Trop2	Basal cells (Goldstein et al., 2008)	Basal, luminal, and neuroendocrine cells in prostatic tubules are regenerated from trophoblast cell surface antigen 2 (Trop2) (hi) basal cells (Goldstein et al., 2008)
β-catenin	Basal cells (Lu and Chen, 2015)	The preferential expression of β-catenin in the CD44^+^ PCa cells will endow them with certain CSC properties (Patrawala et al., 2006)
ERα	Basal cells (Shen et al., 2019)	Estrogen receptor alpha (ERα) has a key role in coordinating CSCs to control prostate organ development (Shen et al., 2019)
CD44	Basal and luminal cells (Wang et al., 2009)	CSC markers aldehyde dehydrogenase^++high^ (ALDH^++high^) and CD44 α2-integrin^+high^ in primary PCa present a basal cell phenotype while showing a luminal progenitor phenotype after ADT treatment (Wang et al., 2009)
ALDH		
α2-integrin		
CD117	Basal and luminal cells (Harris et al., 2021)	CD117 (C-Kit) is a PCSC marker (Leong et al., 2008)
Sca-1	Basal and luminal cells (Xin et al., 2005)	Sca-1 is enriched in murine prostate cells capable of regenerating tubular structures containing basal and luminal cell lineages (Xin et al., 2005)
SOX2	Basal and luminal cells (de Wet et al., 2022)	TMPRSS4 mediates CSC features through the upregulation of SOX2 (Lee et al., 2021)
Nanog	Basal and luminal cells (Jeter et al., 2009)	Nanog protein level is enriched in CSC populations (Jeter et al., 2009)
CK5	Basal and luminal cells (Tang, 2022)	The basal and luminal progenitor cells are frequently double-positive for CK5 (KRT5) (Tang, 2022)
CK8	Luminal cells (Tang, 2022)	The luminal cell layer contains differentiated CK8^+^/CK18^+^/AR^+^/PSA^+^/CD26^+^ luminal cells and the luminal progenitor cells (CK5^+^/CK19^+^) (Tang, 2022)
CK19		
CK18		
CD26		
OCT3/4	Luminal cells (Costa et al., 2019)	POU class 5 homeobox 1 (OCT-3/4) is expressed in some stem-like cancer cells (Patrawala et al., 2006)
DLL4	Luminal cells (Zhang et al., 2016a)	DLL4 facilitates stem cell self-renewal and blood vessel formation (Iyer et al., 2013)
Tacstd2	Luminal cell (Guo et al., 2020)	The results characterize Dist-Luminal-C cells as Tacstd2, CK4, and PSCA expressions and reveal their contributions as drivers of distal prostate luminal lineages (Guo et al., 2020)
CK4		
PSCA		
BMI-1	Luminal cells (Yoo et al., 2016)	B-cell-specific Moloney murine leukemia virus insertion region 1 (BMI-1) often overexpresses and participates in stem cell self-renewal and tumorigenesis of prostate cancer (Li et al., 2017b)
NKX3.1	Luminal cells (Banerjee et al., 2023)	Castration-resistant Nkx3.1-expressing cells are the cells of origin in some types of prostate cancer (Banerjee et al., 2023)
EZH2	Luminal cells (Yuan et al., 2020)	Enhancer of zeste homolog 2 (EZH2) is a common CSC marker (Verma et al., 2023)
ABCG2	Luminal cell (Sabnis et al., 2017)	Inhibiting the adenosine triphosphate (ATP)-binding cassette efflux transporter G2 (ABCG2)-mediated androgen efflux forces the PCSCs to undergo an AR-modulated differentiation to an ADT-sensitive luminal phenotype (Sabnis et al., 2017)
Cripto-1	Secretory (Verma et al., 2023)	Prostate tumor cell lines contain a presumptive cancer stem cell population marked by SUZ-12 and Cripto-1 (TDGF1) (Cocciadiferro et al., 2009)
SUZ12	Intracellular (Verma et al., 2023)	
E-cadherin	EMT (Wolf et al., 2022)	The ability to modulate E-cadherin is the key permissive factor enabling CSC invasion *in vitro* (Wolf et al., 2022)
CD51	Cell surface (Sui et al., 2018)	CD51 (integrin alpha V) could be a functional surface marker for PCSCs (Sui et al., 2018)

### 2.2 Luminal cells

The findings regarding cells-of-origin for NEPC are intriguing. Ci et al., 2020. established a patient-derived xenograft (PDX) model of adenocarcinoma (LTL331)-to-NEPC (LTL331R) transdifferentiation to support a basal progenitor cell model (Ci et al., 2020). Dong et al. (2020) employed the single-cell RNA sequencing detecting transcriptomes of six CRPC needle biopsies, which provided direct evidence of the cellular states underlying luminal–neuroendocrine transdifferentiation. Notably, this transdifferentiation has never been revealed in normal prostate development. In addition, basal cell marker p63 was considered indispensable for prostate development (Goldstein et al., 2008). The next year, explants from p63 null mice could form prostate tissue in the absence of basal cells, supporting the necessity of luminal progenitor cells (Wang et al., 2009). Furthermore, a growing body of evidence indicated that cancers could be driven by tumorigenic luminal cells without initiating basal cells, and murine lineage-tracing experiments also presented luminal-to-basal differentiation (Karthaus et al., 2014).

The controversies are worth pondering. If basal stem cells could represent a cell type of origin, one must wonder why basal or squamous cell carcinomas account for a small proportion of PCa phenotypes (Ali and Epstein, 2007). Given culture condition scarcity, prostatic gland architecture could not be realistically reconstituted. It has remained challenging to determine whether these transitions apply to humans in the absence of a 3D culture system. In terms of organoids of luminal and basal cells, Karthaus et al. (2014) proved that luminal-derived organoids more closely resemble prostate glands. Tang’s research also confirmed luminal progenitor cell (LP) as a preferred cell of origin for PCa (Tang, 2022). Furthermore, Gao’s group brought out a novel insight into tracking of cells-or-origin for mouse prostate. Briefly, they characterized Dist-Luminal-C cells as tumor-associated calcium signal transducer 2 (Tacstd2), CK4, and prostate stem cell antigen (PSCA) expression and revealed its contribution as the driver of distal prostate luminal lineages (Guo et al., 2020). In addition, not only basal compartment but also luminal markers such as NKX3.1, CK18, CK8, CD26, OCT3/4, and delta-like ligand 4 (DLL4) et al. have been demonstrated to be co-expressed with the CSCs (details are shown in Table1) (Wang et al., 2009; Zhang D. et al., 2016; Park et al., 2016; Costa et al., 2019). Most notably, basal stem-like cells have been suggested to be the cell of origin in primary prostatic tumors, while only stem-like cells with luminal phenotype reinitiated CRPC deterioration after androgen ablation (Wang et al., 2009; Germann et al., 2012). For example, CSC markers (ALDH^++high^ CD44 α2-integrin^+high^) in primary PCa presented a basal cell phenotype while showing a luminal progenitor phenotype after ADT treatment. One explanation is that primary treatment-induced lower AR level results in an AR^low^ stem-like luminal cell (Zhang B. et al., 2016). Meanwhile, luminal progenitor cell plays a significant role in treatment resistance and poor outcomes. Over the course of CRPC progression, significant increases in PSA^−/lo^ PCa cells with LP characteristics and human LP markers (i.e., CD38^low^ and ALDH^hi^ CD44 α2β1) have been demonstrated (Zhang D. et al., 2018; Verma et al., 2023; Gorodetska et al., 2024). We propose that low-grade prostate tumors are driven by basal cells, but tumorigenic luminal and LP cells rapidly expand in CRPC.

### 2.3 Others

Several studies have shown that PCSCs could originate from cancerous cells (i.e., inflammatory cells and stromal cells) (Banerjee et al., 2023). This viewpoint could explain why PCSCs have the renewal capacity to achieve malignant transformation where the differentiated cells present accumulative mutations (Banerjee et al., 2023). Herein, inflammation-induced alterations not only cause epithelial lineage differentiation but also promote oncogenic signaling to induce tumor initiation. Many studies have shown that the stem phenotype of advanced PCa was intimately associated with epithelial–mesenchymal transition (EMT), which was derived from stromal cells in the tumor microenvironment (Chen et al., 2020). In addition, observations suggested enhancer of zeste homolog (EZH2)- and cancer-associated fibroblasts (CAF)-mediated EMT resulted in the enrichment of CSC-like properties (Giannoni et al., 2010; Yamada and Beltran, 2021). Additionally, various signaling pathways involved in the progression and therapy resistance, such as Notch, Wingless (Wnt)/β-Catenin, Hedgehog, Hippo, Ras/mitogen-activated protein kinase (MAPK), Janus kinase (JAK)/signal transducer and activator of transcription (STAT), phosphoinositide 3 kinase (PI3K)/protein kinase B (AKT)/mammalian target of rapamycin (mTOR), epidermal growth factor receptor (EGFR), and hypoxia-inducible factor (HIF), have been reported to drive CSC emergence (details are shown in Table 2) (Meisel et al., 2020; Wu et al., 2020; Yang et al., 2020; Ramesh et al., 2023; Verma et al., 2023).

**TABLE 2:** List of drugs for CSC targeted therapy under clinical trials.

Drug	Target	Associated pathway	Clinical trial number	Approved stage	Reference
Exelixis (XL147)	PI3K	PI3K/AKT/mTOR pathway	NCT00704392	Phase I	Sarker et al. (2009), Chang et al. (2015), Hotte et al. (2019), Ranjbar et al. (2023)
Pictilisib (GDC-0941)			NCT01918306	Phase II	
NVP-BEZ235			NCT01717898	Phase II	
PX-866			NCT01331083	Phase II	
Buparlisib (BKM120)			NCT01385293	Phase II	
Idelalisib (Zydelig)			NCT03878524	Phase I	
Everolimus	mTOR		NCT03014297	Phase I	
Temsirolimus			NCT02093598	Phase II	
Ridaforolimus			NCT01380184	Phase I	
AZD8186, AZD2014			NCT01884285	Phase I	
Perifosine	AKT		NCT00590954	Phase II	
GSK690693			NCT00493818	Phase I	
MK2206			NCT01251861	Phase II	
CI-1040	MEK	RAS/MAPK pathway	NCT00034827	Phase II	Santarpia et al. (2012)
ARRY-438162			NCT00959127	Phase I	
AZD6244/ARRY-142886			NCT01605916	Phase I	
Refametinib (BAY 86-9766)			NCT00785226	Phase II	
Trametinib (GSK1120212)			NCT02881242	Phase II	
TAK-733			NCT00948467	Phase I	
Cobimetinib (GDC-0973)			NCT03878524	Phase I	
AZD8330/ARRY-424704			NCT00454090	Phase I	
Avutometinib (RO5126766)			NCT00773526	Phase I	
RO4987655			NCT00817518	Phase I	
Pimasertib (AS703026)			NCT01713036	Phase I	
LErafAON	RAF		NCT00024661	Phase I	
Vemurafenib (PLX4032)			NCT03878524	Phase I	
Raf-265			NCT01352273	Phase I	
XL281 (Exelixis)			NCT00451880	Phase I	
Dabrafenib (GSK2118436)			NCT02465060	Phase II	
Vismodegib (GDC-0449)	SMO	Hedgehog pathway	NCT01163084	Phase II	Karlou et al. (2010), Tong et al. (2018), Saad et al. (2019)
Sonidegib (LDE-225)			NCT02111187	Phase I	
Taladegib (LY2940680)			NCT01226485	Phase I	
TAK-441			NCT01204073	Phase I	
Itraconazole	Hh pathway		NCT01787331	Phase II	
Vantictumab (OMP-18R5)	Fzd7	WNT pathway	NCT01345201	Phase I	Worthmuller and Ruegg (2020), Verma et al. (2023)
Ipafricept (OMP-54F28)	Fzd8		NCT01608867	Phase I	
Rosmantuzumab (OMP-131R10)	R-spondin3		NCT02482441	Phase I	
Foxy-5	Wnt-5a		NCT03883802	Phase II	
PRI-724	β-catenin-CBP		NCT01302405	Phase I	
PRI-724			NCT01764477	Phase I	
SM08502	CLK		NCT03355066	Phase I	
Wnt974 (LGK974)	Porcupine		NCT01351103	Phase I	
ETC-159			NCT02521844	Phase I	
RXC004			NCT03447470	Phase I	
CGX1321			NCT02675946	Phase I	
Aspirin	Wnt6		NCT00316927	Phase III	
Niclosamide	Wnt Wnt		NCT03123978	Phase I	
Celecoxib			NCT01220973	Phase II	
Capsaicin			NCT02037464	Phase II	
Verteporfin	YAP	Hippo pathway	NCT03067051	Phase II	Coffey (2021)
Statins			NCT05586360	Phase II	
Dasatinib	Tyr		NCT00439270	Phase II	
Apatorsen	HSP27		NCT01120470	Phase II	
Crizotinib	ALK		NCT02207504	Phase I	
Alectinib			NCT05238831	Early Phase I	
Pacritinib (SB1518)	JAK2	JAK/STAT pathway	NCT04635059	Phase 2	Kroon et al. (2013), Hall et al. (2020), McLornan et al. (2021), Banerjee et al. (2023)
Fedratinib (SAR302503)			NCT01836705	Phase I	
Momelotinib (GS-0387, CYT-387)	JAK1 and JAK2		NCT02244489	Phase I	
Ruxolitinib			NCT00638378	Phase II	
Tofacitinib	JAK3		NCT04034238	Phase I	
Itacitinib	JAK1		NCT02559492	Phase I	
Siltuximab (CNTO 328)	IL-6		NCT00433446	Phase II	
Tocilizumab			NCT03821246	Phase II	
RO4929097	γ-secretase	Notch pathway	NCT01200810	Phase II	Groth and Fortini (2012), Kanwal et al. (2020)
MK-0752			NCT01295632	Phase I	
PF-03084014			NCT02299635	Phase II	
OMP-59R5	Notch2 and 3		NCT01277146	Phase I	
Demcizumab (OMP-21M18)	DLL4		NCT02722954	Phase I	
PAN-301-1	ASPH		NCT03120832	Phase I	
Lapatinib (GW572016)	EGFR	EGFR pathway	NCT00246753	Phase II	Sridhar et al. (2010), Ojemuyiwa et al. (2014), Wang et al. (2018)
Erlotinib			NCT00272038	Phase II	
Gefitinib			NCT00483561	Phase II	
C225-ILS-DOX			NCT02833766	Phase II	
Imatinib	PDGFR		NCT00424385	Phase I	
Sunitinib (SU11248)	VEGFR		NCT00299741	Phase II	
Cediranib (AZD2171)			NCT00436956	Phase II	
Sorafenib (BAY 43-9006)	Src		NCT00090545	Phase II	
Dasatinib			NCT00439270	Phase II	
Cabozantinib	VEGFR2		NCT01834651	Phase II	
MM-302	HER2		NCT02213744	Phase III	
Tasquinimod	TSP1	HIF pathway	NCT02396368	Phase I	Olsson et al. (2010)
Digoxin	HIF-α		NCT01162135	Phase II	Lin et al. (2009)
Celecoxib	SOX2		NCT00073970	Phase II	Sooriakumaran et al. (2009)
Metformin	AMP-Kinase	EMT	NCT01620593	Phase II	Chaves et al. (2021)
Adavosertib	WEE 1		NCT03385655	Phase II	
Romidepsin	HDACs		NCT00106418	Phase II	
Panobinostat			NCT00667862	Phase II	
Pracinostat			NCT01075308	Phase II	
Vorinostat			NCT00330161	Phase II	
Phenylbutyrate			NCT00006019	Phase II	
Tazemetostat	EZH2		NCT04179864	Phase II	
CPI-1205			NCT03480646	Phase II	
Azacitidine	DNMTs		NCT03572387	Phase II	
Decitabine			NCT02649790	Phase II	

Isolating cells with tumor-initiating and stem-like properties like PCSCs presents undeniable challenges. Utilizing specific markers expressed by PCSCs can offer solutions. Techniques such as fluorescence-activated cell sorting (FACS) and magnetic-activated cell sorting (MACS) can effectively isolate and purify PCSCs based on known surface markers (Banerjee et al., 2023). For instance, selecting for CD44^+^α2β1^-/lo^ cells has been proposed as a representation of PCSCs (Patrawala et al., 2007). Additionally, nuclear staining dyes like Hoechst 33,342 and Rhodamine 123 can aid in isolating PCSCs. It is reported that strategically repeated chemotherapy and radiotherapy could maintain cell populations of therapy-resistant phenotypes and provide favorable conditions for PCSC proliferation. The sphere formation assay has been suggested as another option. Spheres derived from PCSCs can be further characterized (Verma et al., 2023). Thus, PCSCs can be isolated either by selecting marker-based populations or by inducing cell de-differentiation.

## 3 Therapeutic strategies targeting PCSCs

Current treatments for PCa, such as ADT, chemotherapy, and radiation, are designed to eliminate large numbers of conventional tumor cells but do not appear to be effective against drug-resistant PCSCs. Therefore, therapies targeting PCSCs are emerging as promising approaches. These approaches focus on PCSC-related pathways, the PCSC microenvironment, miRNA, and immunotherapy. In this context, several inhibitors have been reported in clinical trials or are undergoing clinical trial evaluation (details are shown in Table 2).

### 3.1 Targeting PCSC-related signaling pathways

#### 3.1.1 PI3K/AKT/mTOR

PI3K, frequently activated in PCa, stimulates mTOR through activated AKT. Recent discoveries indicated that the intricate crosstalk within the PI3K/AKT/mTOR pathway could facilitate tumor formation, enhance CSC properties, and increase therapeutic resistance (Verma et al., 2023). To date, several inhibitors targeting the PI3K/AKT/mTOR pathway have been evaluated in phase I or II clinical trials (details are shown in Table 2). These inhibitors could also be used with chemo- or radiotherapy to restore the sensitivity of CRPC patients to traditional treatments (Bitting and Armstrong, 2013). In PTEN-loss models, the inhibition of AR could activate the PI3K/AKT pathway and vice-versa. To address the problem, a PI3K inhibitor (such as PX-866) was designed to target CRPC patients, which had a beneficial effect and overcame resistance (Hotte et al., 2019). However, the dual PI3K and mTOR inhibition might cause unpredictable toxicity in patients with mCRPC (Wei et al., 2017).

#### 3.1.2 RAS/MAPK

MAPK signaling is reported to be responsible for stem characteristics in PCSCs, and phosphorylation events play critical parts in tumorigenesis (Santarpia et al., 2012). Hindering MAPK via targeted inhibitors has been an applicable model for cancer therapeutics. Abnormal activation of the RAS-RAF-MEK-ERK-MAPK (RAS-MAPK) pathway promotes CSC self-propelling and poses a second hit to an alteration of the PTEN/PI3K/AKT axis (Santarpia et al., 2012). MAPK kinase inhibitor PD098059 restored the growth inhibitory role of TGF-β1 in PCa, which carried an oncogenic mutation in RAS (Park et al., 2000). Although PD098059 and PD325901 have been demonstrated to be effective in mouse studies, they have not been targeted for clinical development (Mukhopadhyay et al., 2007; Mulholland et al., 2012). PD184352 (CI-1040) has been evaluated in phase I clinical trials but not yet verified in phase II trials (Papatsoris et al., 2007). In addition, drugs that obstruct the RAS/MAPK pathway might exhibit widespread mechanism-induced toxicities.

#### 3.1.3 Hedgehog

Emerging studies have demonstrated that the abnormal involvement of Hedgehog signaling was accountable for PCSC maintenance. Recently, preclinical studies showed that PCSCs were subjected to Hedgehog-related inhibition (Banerjee et al., 2023). One such Hedgehog receptor smoothened (SMO) inhibitor is GDC-0449, which promotes PCSC apoptosis via GLI-dependent regulation (Tong et al., 2018). A randomized phase I/II trial study explored antihormone therapy together with GDC-0449 to see how well they work in advanced PCa patients, and the results were highly anticipated (NCT01163084). Sonidegib, an SMO inhibitor, underwent a phase I clinical trial in patients with high-risk localized PCa and caused a 2-fold reduction in GLI1 levels (Tong et al., 2018). Other inhibitors of GLI1, such as IPI-269609, GANT61, GANT58, zerumbone, physalin F and physalin B, and SMO inhibitor CUR61414, have not yet been tested in clinical trials (Karlou et al., 2010). Identifying the stages of PCa may provide the most clinical benefit.

#### 3.1.4 Wnt

The Kjd Wnt/β-catenin signaling pathway is one of the vital mechanisms responsible for PCa self-renewal ability, and dysregulation of Wnt signaling increases the proportion of PCSCs (Karlou et al., 2010). An *in vitro* study suggested that capsaicin could be a potential chemotherapeutic drug for CRPC via blocking the Wnt/β-catenin pathway (Ramesh et al., 2023). Accordingly, a phase II trial was designed to determine the chemopreventive properties of capsaicin in PCa patients enrolled in the active surveillance program or patients scheduled to undergo radical prostatectomy (NCT02037464). In addition, agents like aspirin, which has been approved by the FDA, are applied in the clinics (Verma et al., 2023). Consequently, Wnt-related research has been a significant field for the development and application of targeted drugs. The inhibitors targeting the Wnt/β-catenin pathway are classified into non-steroidal anti-inflammatory drugs (ibuprofen and aspirin) and CBP/β antagonists (ICG-001 and NSC668036) (Verma et al., 2023). Meanwhile, Worthmuller and Ruegg (2020) divide Wnt-related agents into ligand/receptor level (vantictumab, ipafricept, etc.), transcriptional level (CWP232291, PRI-724, etc.), and Wnt secretion (WNT974, ETC-15, etc).

#### 3.1.5 Hippo

The Hippo pathway and its core downstream effectors, Yes-associated protein (YAP) and paralog, a transcriptional coactivator with the PDZ-binding motif (TAZ), are crucial for tissue regeneration through the regulation of stem cells (Messina et al., 2023). Inhibition of Hippo remains challenging owing to its complicated regulation and crossing with other pathways. Although the YAP/TAZ targeted therapeutic drug, verteporfin, has been approved by the FDA, its future use for cancer treatment appears to be multimodal, relying on the cellular background (Coffey, 2021). In addition, several FAK inhibitors have been measured in clinical trials with prospective results in PCa. One is apatorsen (OGX427), which could induce tumor regression in preclinical models of metastatic CRPC and has shown encouraging preliminary results in phase II clinical trials (Coffey, 2021).

#### 3.1.6 JAK/STAT

Gene expression profiling of CD44^+^/α2β1^hi^/CD133^+^ primary cancer cells reveals a significant over-representation of the JAK-STAT signaling pathway, indicating aberrant alterations of this pathway in CSCs could accelerate the tumor load (Kroon et al., 2013). Wang et al. (2024) demonstrated that blocking STAT3 via berbamine resulted in downregulation of CSC level and increased drug sensitivity to cabazitaxel. However, there are not yet any clinical trials for berbamine. The blockade of activated STAT3 by another anti-IL-6 antibody, tocilizumab, suppressed the activity of the TAM-stimulated CD44^+^ cells in high-grade diseases (Wan et al., 2014). A phase I trial was aimed to evaluate the safety and efficacy of CC-1 (a dual mode of anticancer action) with prophylactic IL-6R blockade using tocilizumab in CRPC patients after failure of third-line therapy (NCT04104607). The research would help better define the action of CC-1 and identify biomarkers for further clinical development.

#### 3.1.7 Notch

The Notch pathway, which regulates cell fate determination, metastasis, and chemoresistance, has been found to be dysregulated in PCa (Banerjee et al., 2023). One approach involves the exploration of antibodies to obstruct specific Notch receptors, their activating ligands, or other targets of the Notch signaling in tumors (Han et al., 2021). Chemotherapy combined with Notch1 inhibitors is proved to reduce the chemotherapy-enriched CSC population in a complementary manner (Banerjee et al., 2023). Recently, Cui et al. (2015) suggested that Notch blocking via a γ-secretase inhibitor (GSI) named PF-03084014 could slow the growth of tumor cells and reinforce the anti-metastatic effect of docetaxel in PCa *in vivo* and *in vitro*. In contrast, PF-03084014 failed to produce a clinical benefit to CRPC patients owing to its systemic toxicity and off-target effects (Banerjee et al., 2023). Another small-molecule inhibitor of aspartate β-hydroxylase (ASPH), PAN-301-1 vaccine against ASPH has been tested in a phase I clinical trial in PCa patients, indicating that ASPH is a promising target (Kanwal et al., 2020).

#### 3.1.8 EGFR

Rybak et al. (2013) have presented evidence that EGFR signaling promoted maintenance of PCSC-like characteristics, in part by stimulating the MEK-ERK pathway. Inhibition of ERK activation by U0126 treatment and ERK1/ERK2 knockdown could account for a rapid reduction in PCSC propagation (Rybak et al., 2013). Clinically, modulation of the EGFR pathway is correlated with therapeutic efficiency. Recently, there has been a trend in evaluating tyrosine kinase inhibitors (TKIs) that impede angiogenic growth factor targets. A phase II trial tested sorafenib, an oral inhibitor of EGFR, in metastatic CRPC patients. This agent works by blocking radiological progression and, in part, promoting the regression of bone metastases (Antonarakis et al., 2010). Erlotinib is also a selective TKI of EGFR and has moderate activity in chemotherapy-naïve CRPC in combination with chemotherapy (Nabhan et al., 2009). In addition, PCa has upregulation of platelet-derived growth factor receptor (PDGFR), cooperating with the PI3K/AKT pathway. However, the antitumor effect of PDGFR inhibitor imatinib has been disappointing (Antonarakis et al., 2010).

#### 3.1.9 HIF

HIF signaling is activated in PCa in response to hypoxic conditions within the tumor microenvironment. O’Reilly et al. (2019) demonstrated that HIF-2α interacted with SOX2 under long-term hypoxia, promoting stem cell renewal and metastasis of PCSCs. Taken together, these identify HIF and associated pathways as novel cancer drug targets, as well as inhibitors of the hypoxia-response pathway, that are being developed. A phase II clinical trial using oral tasquinimod exhibited moderate activity against mCRPC via upregulation of TSP1, accounting for the downregulation of HIF-1α (Olsson et al., 2010). In addition, camptothecin (CPT), a potent inhibitor of HIF-1α, failed to produce a clinical benefit owing to significant toxicity. Schmidt et al. (2020) designed a nanoparticle–drug conjugate (NDC) of CPT named NLG207 to facilitate drug delivery to tumors. Work on this is ongoing at the National Cancer Institute.

### 3.2 Targeting the PCSC microenvironment

Tumor cells undergo EMT, wherein they lose their epithelial surface markers, most notably E-cadherin, and obtain mesenchymal markers, including vimentin and N-cadherin (Banerjee et al., 2023). Drivers (such as Snail, Twist, and STAT3) and abundant signaling pathways are activated in EMT (Chaves et al., 2021). Given their vital roles in the EMT process, treatments aimed at suppressing specific regulations could provide an approach to achieve the antineoplastic effect. It is already confirmed that miRNAs affected the proportion of PCSCs indirectly via the EMT process. Zhang et al. proved that metformin prevented EMT via microRNA-30a-modulated SOX4 expression (Zhang et al., 2014). However, a phase II trial named “castration compared to castration plus metformin as first-line treatment for patients with advanced PCa” yielded no clinical benefit of adding metformin (NCT01620593). In addition, the abnormality of miR-205 could impede CAF-mediated EMT *in vitro* and *in vivo* (Ramesh et al., 2023).

Anticancer strategies have been developed for CAF, varying from metronomic chemotherapy to immune-based therapies. For instance, a GPR77-neutralizing antibody is demonstrated to be valid for restoring tumor sensitivity to chemotherapy in a PDX model (Fiori et al., 2019). Moreover, tazemetostat (EZH2 inhibitor) hitting the PRC2-mediated EMT is designed to determine the recommended dose of tazemetostat in combination with either enzalutamide or abiraterone/prednisone. This approach is being evaluated in a phase II clinical trial enrolling advanced PCa patients (NCT04179864). Presently, efforts to develop therapeutic agents targeting EMT are in progress, and promising results are within reach.

### 3.3 miRNA therapy

Some miRNAs that are related to good prognosis have been downregulated in CRPC patients. miR-34a, miR-708, miR-143, and miR-145 are negative regulators of CD44 in PCSCs and thus have the potential to serve as therapeutic drugs for advanced PCa patients (Ramesh et al., 2023). In addition, it has been suggested that overexpression of miR-let-7c, miR-101-3p, and miR-138-5p could block the stemness of PCSCs by suppressing EZH2 (Kong et al., 2012; Rizzo, 2021; Ramesh et al., 2023). Subsequently, BR-DIM (metabolite 3,3′-diindolylmethane) is applied to reduce PCSC percentages through EZH2 downregulation (Kong et al., 2012). Mechanistically, miR-7, miR-100, miR-143/miR-145, miR-218, miR-199a-3p, miR-141, and miR-320 suppress PCSCs by targeting the KLF4/PI3K/AKT/p21 pathways, oncogene argonaute 2 (AGO2), OCT4, GLI1, EGFR, actin related protein 2/3 complex subunit 5 (ARPC5), and Wnt/β-catenin, respectively (Rizzo, 2021; Ramesh et al., 2023). For chemotherapy resistance, the expression of miR-125a-3p, miR-34a-5p, miR-204, miR-205, and miR-3 could hamper the enrichment of stem cells and strengthen docetaxel sensitivity in PCa samples, making them ideal therapeutic targets (Rizzo, 2021). In particular, miR-205 also increases radiation sensitivity (El Bezawy et al., 2019). In summary, new therapeutic approaches based on miRNAs might be a good prospect.

### 3.4 Immunotherapy

Recently, increasing numbers of clinical trials have addressed immunotherapy incorporating vaccine-based therapies, immune checkpoint inhibitors (ICIs), and chimeric antigen receptor (CAR)-modified T-cell therapy, which targets CSC-associated tumor antigens. These products are emerging as new therapeutic approaches for advanced PCa patients (Bansal et al., 2021).

#### 3.4.1 Immune checkpoint inhibitors (ICIs)

ICIs present antitumor activities by targeting the dysfunctional immune system, where a T-cell antitumor response is generated (Lentz et al., 2021). Ipilimumab is a humanized anti-CTLA-4 antibody that is expressed on the surface of T lymphocytes (Chen, 2004). Its use to treat PCa is investigational. An early phase I clinical trial is aimed at studying the impact of ipilimumab on the immune system of patients receiving hormone therapy, but subsequent results have not yet been presented (NCT02113657). Examples of other immune checkpoint protein PD-1 inhibitors are nivolumab and pembrolizumab, which restore T cells’ ability to eradicate cancer cells (Bansal et al., 2021). A recent update on a phase II clinical trial confirmed the antitumor activity of pembrolizumab with an acceptable safety and encouraging OS evaluation (NCT02787005) (Antonarakis et al., 2020).

Anti-PD-L1 immunotherapies, such as avelumab and atezolizumab, are also being studied (Bansal et al., 2021). In 2021, an ongoing phase II clinical trial of avelumab was designed to evaluate its effects against PICK-NEPC (NCT03179410). Evaluations of monotherapy and the strategies cooperating ICIs with chemotherapy, radiation, PARP inhibitors, adenosine receptor antagonists, IL-2 agonists, and CD11b agonists are in progress (Bansal et al., 2021). For instance, an investigational immunotherapy of nivolumab in combination with rucaparib, docetaxel, or enzalutamide in mCRPC patients is ongoing (NCT03338790). Fizazi et al. (2022) reported results from cohorts A1 and A2 of CheckMate 9KD that nivolumab plus rucaparib were active in HRD-positive postchemotherapy or chemotherapy-naïve mCRPC groups. Notably, a further step is needed to reveal whether nivolumab supplementary incrementally improves OS versus rucaparib alone (Fizazi et al., 2022). Additionally, an immunosuppressive TME and impaired cellular immunity may impede ICI application in advanced PCa (Bansal et al., 2021).

#### 3.4.2 Vaccine-based therapies

A vaccine based on tumor-associated antigen (TAA) could activate a particular immune response to cancer cells. PCa could express substantial TAA involving PSA, prostate-specific membrane antigen (PSMA), prostatic acid phosphatase (PAP), and PSCA (Bansal et al., 2021). To target these antigens, different forms of PCa vaccines have been developed, such as cellular vaccines, viral vector-based vaccines, polypeptide vaccines, nucleic acid vaccines, and mRNA-based vaccines (Wang et al., 2023). Sipuleucel-T, an FDA-approved autologous cell vaccine, is designed to induce a T-cell-mediated immune response to recombinant PAP (Cha et al., 2020). Currently, related clinical trials have been completed. Phase III (NCT00065442, NCT00005947, and NCT01133704) suggested that sipuleucel-T treatment induced a 3-fold increase in activated T cells from prostatectomy specimens (Ju et al., 2022). It is worth mentioning that the sipuleucel-T treatment can help patients stay where they are rather than fully recovering works to block further deterioration of advanced PCa tumors, not subside. 

PROSTVAC has undergone tests in numerous clinical trials. In a phase II clinical trial (TBC-PRO-002), PROSTVAC was associated with a longer median survival time of 9.9 months in men with mCRPC (Kantoff et al., 2010). Conversely, in low- or intermediate-risk PCa, no differences in postvaccination peripheral T-cell responses were observed (NCT02326805) (Parsons et al., 2023). For cellular vaccines, Wang et al. developed an immunogenic peptide-sensitized dendritic cell (DC)-cytokine-induced killer cell (CIK)-based cell, which manifested an antitumor effect against PCa xenografts derived from the PCSC-enriched prostatospheroids. This therapeutic platform is expected to apply to immunotherapy (Wang et al., 2020).

PCVAC/PCa is another cellular cancer vaccine. Regrettably, the combination therapy of DCVAC/PCa, docetaxel, and prednisone was deemed ineffective in extending OS in patients with mCRPC (NCT02111577) (Vogelzang et al., 2022). In addition, individualized polypeptide vaccine (PPV) stands out, bypassing immune diversity and evading immune tolerance (Wang et al., 2023). Yoshimura et al. (2016) compared clinical outcomes of the treatment with PPV, adding dexamethasone versus dexamethasone alone in 2016, where the PPV group presented longer median OS and progression-free survival (PFS). Of note, the recruited patients in this study were diagnosed in the early stage of CRPC (Yoshimura et al., 2016). Another prostate cancer vaccine, GVAX, has been shown to induce infiltrating immune cells that may promote PD-L1 upregulation (Palicelli et al., 2021). However, the exact efficacy remains to be unveiled.

DNA vaccines could evoke antitumor immune response by changing the sequence of plasmid DNA (Wang et al., 2023). An example is the pTVG-HP vaccine, which encodes the human PAP antigens and is being evaluated in mCRPC trials. Given their instability and inefficiency, the development of mRNA-based vaccines is still in slow progress (Bansal et al., 2021).

#### 3.4.3 Chimeric antigen receptor (CAR)-modified T-cell therapy

CAR-T cell therapy targeting PCSC-associated antigens emerges as a promising therapeutic approach. Despite no results, some phase I clinical trials with PSCA are ongoing to assess the immune activity of PSCA-specific CAR-T cells in patients with mCRPC (NCT03927573 and NCT03873805). Subsequently, BPX-601 acted as a PSCA-directed CAR-T cell and was applied in the clinical trial I/II, in which feasibility, safety, and clinical activity were measured at the recommended dose (NCT02744287). Both BPX-601 and 4-1BB are designed to enhance the immune response of patients with PSCA^+^ mCRPC (NCT03873805). By targeting another well-known antigen (EpCAM), EpCAM-specific CAR-T cell is introduced into human peripheral blood lymphocytes (PBLs) with the strategy of substantially preventing PC-3 growth *in vitro* and *in vivo* (Deng et al., 2015).

Owing to PCSC resistance to fractionated irradiation, which is characterized by high B7-H3 levels, B7-H3 CAR-T cells are demonstrated to support radiation therapy against PCSCs (Zhang et al., 2021). In addition, the CAR-T cell strategy targeting PSMA with lutetium-177 (177Lu-J591) has proven a clinical benefit in phase II clinical trial testing (Tagawa et al., 2013). The results from Frieling et al. (2023) revealed that γδ CAR-T cells targeting PSCA caused a robust regression of established tumors in a preclinical murine model of bone mCRPC. Another novel cell therapy, the tumor-infiltrating lymphocytes (TILs) strategy, has gained striking momentum. Recently, Gao’s group overcame sorafenib resistance to liver cancer by targeting stem-like CCR4^+^ regulatory T cells and inhibiting the maintenance of the TIL-Treg pool (Gao et al., 2022). However, obtaining TILs from PCa patients with poor immunogenicity remains challenging. In 2019, Yunger et al. (2019) managed to expand TILs from eight PCa patients under ADT treatment, supporting the development of prostate-TIL therapy. Experiments based on PC3-bearing humanized immunodeficiency IL2Rγ null (hNSG) mice with an intravenous injection of human CD34^+^ hematopoietic stem cells indicated that the N-cadherin antagonist ADH-1 promoted TIL antitumor responses (Sun et al., 2021). Elevated density of CD8^+^ TILs was demonstrated to improve clinical outcomes from PCa patients undergoing radical prostatectomy (Yang et al., 2021). Although no phase III data have been reported for prostate-TIL products, some clinical trials are recruiting patients. Collectively, CAR-T cells targeting PCSCs and TILs represent promising therapeutic options in the future.

## 4 Conclusion

**FIGURE 1:**
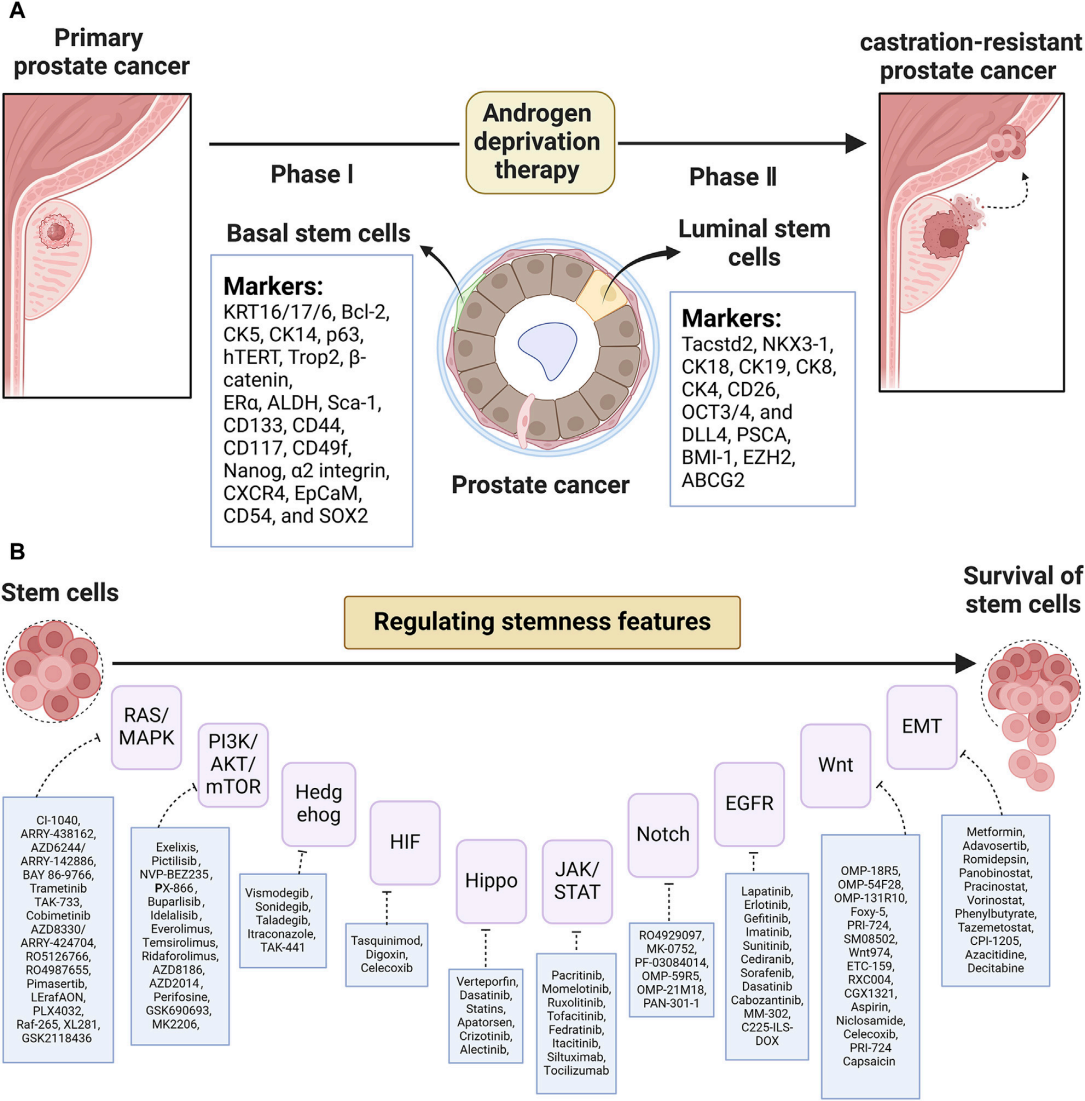
**(A)** List of putative biomarkers for CSCs based on basal and luminal layers and **(B)** CSC-related pathway targeted agents in PCa.

PCSCs are the cancer-initiating cells that play a pivotal role in tumor relapse and therapy resistance. Identifying the characteristics and presence of PCSCs is important to reveal their mechanism and develop targeted therapies against CSC. The establishment of a 3D culture system provides general support for the point that basal stem-like cells are suggested to be the cells of origin in primary prostatic tumors, while stem-like cells with luminal phenotypes reinitiate CRPC relapse after ADT. Additionally, PCSCs could also exist in reprogrammed non-epithelial cancerous cells (i.e., inflammatory and stromal cells). As Figure 1A shows, putative biomarkers for PCSCs from basal (KRT16/17/6, Bcl-2, CK5, CK14, p63, hTERT, Trop2, β-catenin, ERα, ALDH, Sca-1, CD133, CD44, CD117, CD49f, Nanog, α2 integrin, CXCR4, EpCaM, CD54, and SOX2) and luminal (Tacstd2, NKX3-1, CK18, CK19, CK8, CK4, CD26, OCT3/4, DLL4, PSCA, BMI-1, EZH2, ABCG2, etc.) are listed. Of note, PCSC-related therapies concentrating on PCSC-related pathways, the PCSC microenvironment, miRNA, and immunotherapy (see Figure 1B) are valid goals to aim for and also have massive hurdles to overcome. Collectively, based on this review of PCSC characteristics and accessible clinical trials, it is clear that a great need exists for further testing of these targeted therapies.

### 4.1 Limitation

The work has several critical limitations:

1. The description of the isolation and enrichment of PCSCs is limited and warrants a more thorough examination to provide greater insights.
2. Apart from PCSCs, drug resistance in PCa involves factors such as hypoxia, oxidative regulation, EMT, and autophagy. A more extensive discussion is needed.
3. Although numerous clinical studies are underway, their outcomes remain inconclusive. Further monitoring and statistical analysis are warranted.
4. While this work predominantly focuses on the role of signaling pathways in PCSC development, the significance of PCa-related metabolism should also be explored.
